# Ovarian Sex Hormones Modulate Compulsive, Affective and Cognitive Functions in A Non-Induced Mouse Model of Obsessive-Compulsive Disorder

**DOI:** 10.3389/fnbeh.2016.00215

**Published:** 2016-11-09

**Authors:** Swarup Mitra, Cristiane P. Bastos, Katherine Bates, Grace S. Pereira, Abel Bult-Ito

**Affiliations:** ^1^Department of Chemistry and Biochemistry, University of Alaska FairbanksFairbanks, AK, USA; ^2^IDeA Network of Biomedical Research Excellence (INBRE), University of Alaska FairbanksFairbanks, AK, USA; ^3^Núcleo de Neurociências, Departamento de Fisiologia e Biofísica, Instituto de Ciências Biológicas, Universidade Federal de Minas GeraisBelo Horizonte, Brazil; ^4^Department of Biology and Wildlife, University of Alaska FairbanksFairbanks, AK, USA

**Keywords:** compulsive-like behavior, ovariectomy, strain differences, surgical menopause, 17 β-estradiol, progesterone

## Abstract

There is currently a lack of understanding of how surgical menopause can influence obsessions, compulsions and associated affective and cognitive functions in female obsessive-compulsive disorder (OCD) patients. Early menopause in women due to surgical removal of ovaries not only causes dramatic hormonal changes, but also may induce affective and cognitive disorders. Here, we tested if surgical removal of ovaries (ovariectomy, OVX), which mimics surgical menopause in humans, would result in exacerbation of compulsive, affective and cognitive behaviors in mice strains that exhibit a spontaneous compulsive-like phenotype. Female mice from compulsive-like BIG, non-compulsive SMALL and randomly-bred Control strains were subjected to OVX or sham-surgery. After 7 days animals were tested for nest building and marble burying to measure compulsive-like behavior. The elevated plus maze and open field tests measured anxiety-like behaviors, while memory was assessed by the novel object recognition. Acute OVX resulted in exacerbation of compulsive-like and anxiety-like behaviors in compulsive-like BIG mice. No significant effects of OVX were observed for the non-compulsive SMALL and Control strains. Object recognition memory was impaired in compulsive-like BIG female mice compared to the Control mice, without an effect of OVX on the BIG mice. We also tested whether 17 β-estradiol (E2) or progesterone (P4) could reverse the effects of OVX. E2, but not P4, attenuated the compulsive-like behaviors in compulsive-like BIG OVX female mice. The actions of the sex steroids on anxiety-like behaviors in OVX females were strain and behavioral test dependent. Altogether, our results indicate that already existing compulsions can be worsened during acute ovarian deprivation concomitant with exacerbation of affective behaviors and responses to hormonal intervention in OVX female mice can be influenced by genetic background.

## Introduction

Obsessive-compulsive disorder (OCD) is characterized by intrusive thoughts (obsessions) and/or repetitive behaviors (compulsive rituals) in response to the obsessions (American Psychiatric Association, 2013). OCD has a lifetime prevalence of around 2.3% in the United States (Ruscio et al., 2010) and it has been listed as a common mental disorder in adults (Eaton et al., 2008). The obsessive beliefs lead to compulsive symptoms among patients. For example, contamination obsessions can result in compulsive cleaning (Wheaton et al., 2010). Moreover, OCD can negatively impact cognitive and affective functions in humans. Human studies involving neurocognitive tests and image analysis showed impairments in non-verbal (Kashyap et al., 2013), spatial working (van der Wee et al., 2007; Nakao et al., 2009) and visual memories (Dirson et al., 1995). Associated comorbidities like depression (Peris et al., 2010; Remijnse et al., 2013) and anxiety disorders (Nestadt et al., 2001) are also very common in the OCD condition.

Clinical and genetic data for OCD corroborate the hypothesis of sexual dimorphism, which reveals differences in clinical manifestations between males and females (Labad et al., 2008; Torresan et al., 2009). Obsessions for cleaning and compulsive contamination are more prevalent in females than males, while males have higher rates of symmetrical and sexual obsessions when compared to females (Noshirvani et al., 1991; Lensi et al., 1996; Bogetto et al., 1999; de Mathis et al., 2008; Labad et al., 2008). There is also a sex difference to treatment response (Mundo et al., 1999). Women typically have a later onset when compared to men and display a bi-modal distribution with the first peak occurring between 13–16 years of age and the second peak around 22–32 years. These are puberty and child bearing stages in a women’s life, respectively (Brandes et al., 2004) when sex hormone (estrogen and progesterone, P4) levels are known to fluctuate.

It is well established that a plausible cause of OCD is abnormal cortical-striatal-thalamic circuitry activation (Ahmari et al., 2013) and altered serotonergic (Schilman et al., 2010), glutamatergic (Arnold et al., 2004; Egashira et al., 2013; Porton et al., 2013) and GABAergic (Egashira et al., 2013) systems. Interestingly, female hormones, such as estrogen and P4, regulate various neurotransmitter signaling pathways in brain regions implicated in OCD (Dreher et al., 2007; Karakaya et al., 2007; Benmansour et al., 2009; Alonso et al., 2011; Quinlan et al., 2013; Barth et al., 2015). During the estrous phase, circulating estrogen levels are higher and serotonin release is lower in striatal neurons (Yang et al., 2015), while in the frontal cortex, estrogen depletion by ovariectomy (OVX) decreases 5-HT2A receptor density and mRNA levels (Cyr et al., 1998). On the other hand, P4 increases dopamine release mediated by NMDA receptor activation in striatal neurons (Cabrera and Bregonzio, 1996) and decreases NMDA binding density in the frontal cortex after OVX (Cyr et al., 2000). Therefore, ovarian sex hormones may account for the sex differences observed in OCD.

Women are subjected to hormonal fluctuations during their entire life span, which may lead to significant alterations in mood and cognition (Soares and Zitek, 2008). However, remarkable challenges are encountered during the menopause transition due to the natural decline in ovarian function, the primary source of estrogen and P4 (Luine, 2014). Natural menopause is also associated with cognitive deficits and mood disorders (Weber et al., 2012; Dumas et al., 2013). Such dysfunction in mood and cognitive functions has also been reported in women with surgical menopause (Chen et al., 2013; Faubion et al., 2015). Physiological challenges during surgical menopause are much more drastic due to a sudden depletion of ovarian sex steroids as compared to progressive menopause which follows fluctuating patterns of steroid levels (Bachmann, 2001; Rodriguez and Shoupe, 2015; Rodríguez-Landa et al., 2015). This results in greater predisposition to mood and anxiety disorders when compared to natural menopause (Rodríguez-Landa et al., 2015).

The impact of acute ovarian dysfunction during surgical menopause on compulsive behaviors and comorbid affective behaviors in females are currently poorly understood. In addition to younger women, one out of eight women after the age of 55 undergoes bilateral oophorectomy (surgical removal of ovaries) before reaching natural menopause due to benign diseases, prophylaxis against cancer and autoimmune disorders (Shuster et al., 2010; Erekson et al., 2013; Cox and Liu, 2014). Existing studies have investigated obsessions and compulsions only during and after progressive menopause with contradictory evidence (Vulink et al., 2006). One such study showed that, OCD is not a rare comorbidity during post menopause (Uguz et al., 2010), while another study demonstrated that the symptoms are more related with menarche and decreases during menopause (Guglielmi et al., 2014).

In animal studies, acute administration of estradiol (E2) in pre-pubertal female rats exerted an anti-compulsive-like effect (Flaisher-Grinberg et al., 2009), while male mice with estrogen deficiency (aromatase enzyme knockout) displayed compulsive-like behavior (Hill et al., 2007). In OVX rats, concurrent administration of E2 and P4 was able to reduce compulsive-like lever pressing behavior (Fernández-Guasti et al., 2006). Most of these studies were conducted on induced (drug or gene knockouts) models and did not investigate the associated comorbidities like anxiety and cognitive impairments, while only one study looked at the effect of P4 and E2 in the ovariectomized condition (Fernández-Guasti et al., 2006). Moreover, though OCD has a compelling genetic basis (Nestadt et al., 2010) the role of genetic background in influencing steroid actions in OCD condition during menopause has never been explored.

How do already existing compulsions in females get affected during acute sex hormone deprived conditions when compared to non-compulsive females are not clearly known. The co morbid anxiety and cognitive functions associated with OCD during such a physiological state and the role of genetic background in influencing steroid actions demands investigation. According to Maio et al. (2014), our mice developed through selective breeding for phenotypes of increased or decreased amounts of compulsive-like behavior can be a heuristic tool for studying OCD, especially the replicate BIG strains (BIG1 and BIG2). An unpublished study from our lab has shown that there is variation in compulsive-like and affective behaviors between the two replicate BIG strains that mimics heterogeneity as seen in subgroups of OCD patients. This study however did not look into hormonal deprivation and manipulations. We therefore investigated the hypothesis that acute deprivation of estrogen and P4 through OVX for 7 days will increase the compulsive-and anxiety-like behavior and impair novel object recognition memory in compulsive-like mouse strains. We also hypothesized that the administration of estrogen (E2) and P4 will attenuate the exacerbation in compulsive-like, anxiety-like and cognitive behaviors in compulsive-like strains. Though rodents do not have menopause, surgical removal of the ovaries can cause depletion of E4 and P4 (Kato et al., 2013). We therefore used bilateral OVX as the sex hormone deprived surgical menopause model to achieve the experimental endpoints in this study.

## Materials and Methods

The University of Alaska Fairbanks Institutional Animal Care and Use Committee approved the animal care and experimental procedures (IACUC assurance numbers 568518 and 631126).

### Mouse Husbandry

All mice were raised in polypropylene cages (27 cm × 17 cm × 12 cm) and provided with wood shavings under a 12:12 light-dark cycle at 22 ± 1°C. Weaning of the pups was conducted at 19–21 days of age. All mice were housed with same-sex and same-strain littermates until the end of all the experiments. All mice were singly housed just before the behavioral assessments and were returned to their home cages with their littermates following each test. Food (Masuri Rodent Diet #5663, Purina Mills, LLC, St. Louis, MO, USA) and water were available *ad libitum*.

### Experimental Subjects

The mouse model of OCD used for this study was developed from house mouse (*Mus musculus*) strains bidirectionally selected for nest-building behavior (Lynch, 1980; Bult and Lynch, 2000). The stock population for the original selection experiment (Lynch, 1980) was a cross among eight inbred strains, i.e., A, AKR, BLB/c, C3H/2, C57BL, DBA/2, Is/Bi, and RIII, to yield the HS/Ibg outbred strain (McClearn et al., 1970; Lynch, 1980). This resulted in two BIG strains (BIG1 and BIG2) that use a forty-fold larger amount of cotton for their nest than the two SMALL strains (SML1 and SML2) and two randomly-bred control strains (C1 and C2) that show intermediate levels of nesting (Lynch, 1980; Bult and Lynch, 2000). The BIG strains engage in excessive and repetitive nest building (considered to be homologous to hoarding in humans; Warneke, 1993) and marble burying behavior which is dose-dependently attenuated by fluoxetine and clomipramine, but not desipramine, treatment, making the BIG mice a novel non-induced model for OCD (Greene-Schloesser et al., 2011).

For the OVX study, female mice (*Mus musculus*) of six different mouse strains i.e., two each of compulsive-like strains (BIG1 and BIG2), randomly-bred Control strains (C1, C2) and SMALL (SML1, SML2) strains, were used. For the hormone replacement studies in OVX females, only compulsive-like BIG1 and BIG2 female strains were used. All mice were 80–90 days of age during testing. All data were collected by an individual blinded to the outcome of the study.

### Surgical Procedures

For the OVX study, animals were divided into two groups for each strain. One group was sham operated while the other group was OVX (removal of ovaries). All animals in the hormone replacement studies were OVX. For the surgical procedures, females were exposed to isofluroane (4% induction and flow rate of 1.5–2 L/min) anesthesia. Abdominal incisions were made longitudinally and bilaterally in the region below the last lumbar vertebra. The ovary, oviduct and top of the fallopian tubes were tied and removed in the OVX group. For the sham-operated mice, the procedure remained the same except that the ovaries were not removed but only identified (Fonseca et al., 2013). The abdominal wall and the skin were sutured as described by Capettini et al. (2011). All animals were provided ibuprofen in the drinking water 24 h prior to surgery and maintained for 3–4 days post surgery as needed for pain management.

### Hormone Administration

#### E2 Administration

For the E2 administration study, BIG1 and BIG2 females were subdivided into two treatment groups: vehicle and E2 (*n* = 12 females per group). Seven days after OVX, the vehicle group received a single subcutaneous injection of corn oil while the E2 group received 0.1 mg/kg (acute dosage of E2 produces comparable proestrus levels (Walf et al., 2006)) of E2 (Sigma, St. Louis, MO, USA) in corn oil 44 h before behavioral assessments (compulsive and anxiety tasks; Walf et al., 2008b). For the object recognition task, the mice were injected immediately after the training session and were tested 4 h later (Walf et al., 2008a). A total gap of 5 days between each behavior was employed.

#### P4 Administration

For the P4 administration study, BIG1 and BIG2 females were subdivided into vehicle and P4 groups (*n* = 9 females per group). Following 7 days of OVX, the vehicle group received corn oil while the P4 group received 4 mg/kg of P4 1 h before behavioral testing. For the object recognition task, the mice were injected immediately after the training session and the test was performed 4 h later (Walf et al., 2008a). A gap of 3 days between the end of each behavioral test and the next injection was employed. An acute dosage of P4 used in this study approximates circulating and central P4 levels observed during the proestrus phase (Walf et al., 2006).

### Plasma Steroid Levels

To establish that acute OVX leads to depletion of E2 and P4 plasma E2 and P4 levels were determined in plasma samples (*n* = 5–7 per group) of OVX and sham operated compulsive-like BIG female strains (BIG1 and BIG2). All samples were assayed in duplicates using Cayman ELISA kits (Ann Arbor, MI, USA) as per the manufacturer’s instructions. Data collection was accomplished with a Biotek EL808 spectrophotometric plate reader and analyzed by Prism software.

### Compulsive-Like Behaviors

#### Nest-Building

Nest-building behavior was used to assess the compulsive-like phenotype of the female mice (Greene-Schloesser et al., 2011). All mice were housed individually and were allowed to access a pre-weighed cotton roll placed in the cage top food hopper. The amount of cotton used by the mice after 24 h was determined by weighing the cotton roll. As all other behavioral assessments in the P4 administration experiment were performed after 1 h of P4 administration, nest building was measured for 1 h and 24 h of cotton availability, starting 1 h after the injection, to be able to capture the short-term effects of P4 and also to be able to compare this behavior to the 24-h nesting score of the E2 administration experiment.

#### Marble Burying

The marble-burying test was also used to measure compulsive-like behavior (Takeuchi et al., 2002; Thomas et al., 2009; Greene-Schloesser et al., 2011; Angoa-Pérez et al., 2013). All mice were individually introduced to a polypropylene cage (37 cm × 21 cm × 14 cm) containing 20 glass marbles (10 mm in diameter) evenly spaced on 5 cm deep wood shavings firmly pressed into a bedding without access to food or water for 20 min. The total number of marbles buried at least 2/3 in the 20-min period was quantified as compulsive-like digging behavior (Greene-Schloesser et al., 2011). After the 20-min test, the animals were returned to their home cages with littermates.

### Anxiety-Like Behaviors

#### Open Field

The open field test was performed to evaluate anxiety-like behavior in female mice (Crawley, 1985; Meerlo et al., 1999). Female mice were singly housed outside the testing room just prior to testing. The open field apparatus consisted of an open field arena (40 cm × 40 cm × 30 cm). For testing, animals were placed in the center of the field and allowed to explore the arena for 3 min. Entries into the central square (20 cm × 20 cm) (Greene-Schloesser et al., 2011) were recorded by ANYMaze video tracking system (Stoelting Co., Wood Dale, IL, USA). Total number of line crossings was also assessed for sham and OVX strains. The apparatus was cleaned before each test.

#### Elevated Plus Maze

Anxiety-like behavior was further substantiated by the elevated plus maze test. The plus maze apparatus consisted of two open arms (5 cm × 40 cm) and two closed arms (5 cm × 40 cm × 20 cm) at right angles to each other. Each mouse was placed in the central square facing an open arm and was allowed to explore the maze for 5-min duration (Frye et al., 2008). The time spent on the open arms was determined by the ANYMaze video tracking program (Stoelting Co., Wood Dale, IL, USA). The maze was cleaned before each test.

### Novel Object Recognition Test

The novel object recognition test was performed to measure object recognition memory (Antunes and Biala, 2012). Mice were allowed to explore the open field arena (40 cm × 40 cm × 30 cm) without any objects for 3 min during the habituation phase on day 1. Twenty-four hours later on day 2, the training session was performed and mice were introduced to two similar objects (plastic toys) within a 5 cm distance in the open field arena for 3 min. Mice were then taken out of the arena and returned to their home cages. After 4 h, one of the objects was replaced with a novel object of different shape and size. Animals were then reintroduced into the arena and allowed to explore the objects for 3 min in the testing phase. Time spent exploring the familiar and novel objects was recorded with ANYMaze video tracking software (Stoelting Co., Wood Dale, IL, USA). The preference of one object over another was assessed through the Recognition Index (RI: time spent on novel object divided by the time spent on novel and familiar object together; Fonseca et al., 2013).

### Statistical Analysis

All data were analyzed using Statistical Analysis System (SAS) software. A general linear model (GLM) repeated analysis of variance (ANOVA), with strain (BIG, SMALL, Control), replicate nested within strain (1, 2), treatment (OVX, sham), and strain by treatment interaction effects was used to statistically evaluate the effects of OVX on nest building behavior (grams of cotton), marble burying behavior (number of marbles buried), open field behavior (time in seconds in center), elevated plus maze behavior (time in seconds on open arms), and novel object recognition memory (RI). If the replicate nested within strain effect was significant, the strain effect was tested over the replicate effect. If the replicate effect was not significant, the strain effect was tested over the error term.

A GLM ANOVA, with treatment (OVX, sham), strain (BIG1, BIG2), and replicate by treatment interaction effects, was used to statistically evaluate the effects of OVX on E2 (pg/mL) and P4 (ng/mL) plasma levels.

A GLM ANOVA, with treatment (E2, vehicle or P4, vehicle), strain (BIG1, BIG2), and strain by treatment interaction effects, was used to statistically evaluate the effects of females, sex hormone replacement in compulsive-like OVX females on nest building behavior (1 and 24 h nesting scores), marble burying behavior, open field behavior, elevated plus maze behavior and novel object recognition memory.

When significance was found appropriate pairwise comparisons were performed using the studentized range test. The nesting scores were square root transformed to obtain a more normal distribution (Bult and Lynch, 1996, 1997, 2000), while the data are presented as non-transformed nesting scores.

## Results

### Acute OVX Increased Compulsive-Like Behavior in Compulsive-Like BIG Strains

#### Compulsive-Like Nesting

**Figure 1:**
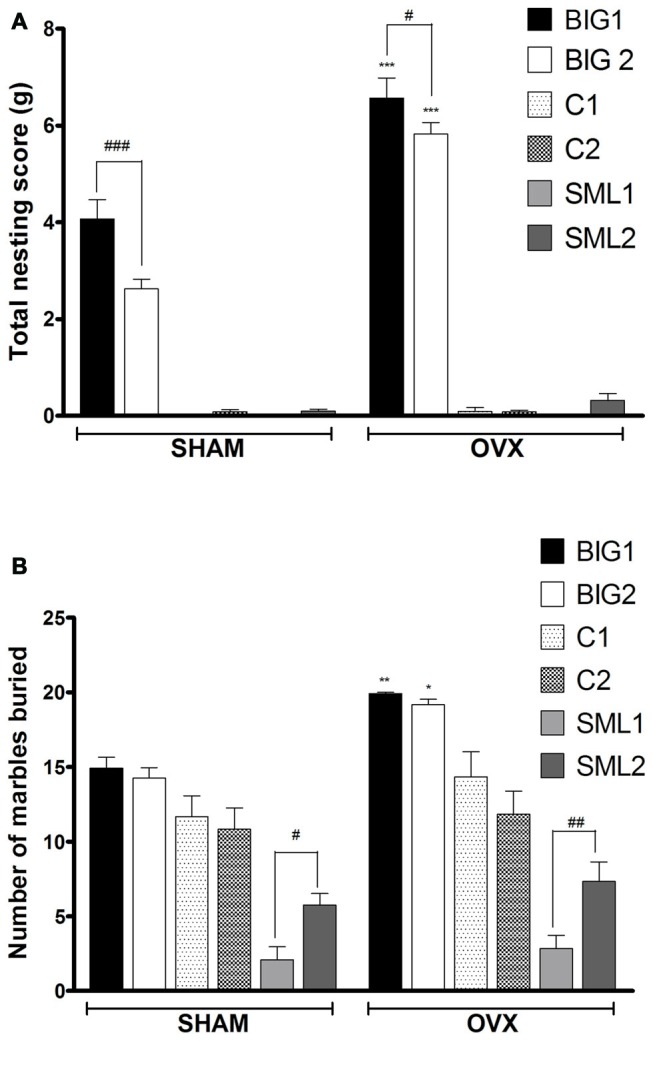
**Compulsive-like behavior in BIG, SMALL and Control strains.** The data represent the mean (± SEM) for **(A)** nesting score in grams between 0–24 h in nest-building test and **(B)** number of marbles buried in marble burying test of the two replicates of the BIG, SMALL and Control strains. *(*p* < 0.05), **(*p* < 0.001) and ***(*p* < 0.0001) indicates significant differences between sham and ovariectomy (OVX) groups. ^#^(*p* < 0.05), ^##^(*p* < 0.001) and ^###^(*p* < 0.0001) indicate significant differences between replicate strains.

Acute OVX in compulsive-like BIG1 (*post hoc* *t*_(22)_ = 8.983, *p* < 0.0001) and BIG2 (*post hoc* *t*_(22)_ = 11.51, *p* < 0.0001) females resulted in significant increases of nesting behavior when compared to the sham operated ones (*F*_(1,134)_ = 77.60, *p* < 0.0001). No significant increases of compulsive-like nesting were observed in the SMALL (SML1: *post hoc* *t*_(21)_ = 0.0045, *p* > 0.99; SML2: *post hoc* *t*_(22)_ = 0.794, *p* > 0.43) and Control (C1: *post hoc* *t*_(22)_ = 0.270, *p* > 0.78; C2: *post hoc* *t*_(22)_ = 0.0150, *p* > 0.98) OVX strains when compared to their sham operated controls (Figure 1A), which explains the significant strain by treatment interaction effect (*F*_(2,134)_ = 65.91, *p* < 0.0001). The BIG strains built bigger nests than the SMALL and Control mice (*F*_(2,*3*)_ = 70.84, *p* < 0.0001). The replicate nested within strain effect was also significant (*F*_(3,134)_ = 10.59, *p* < 0.0001), predominantly due to the BIG1 females building bigger nests than the BIG2 females (sham: *post hoc* *t*_(22)_ = 5.188, *p* < 0.0001; OVX: *post hoc* *t*_(22)_ = 2.666, *p* < 0.05).

#### Compulsive-Like Marble Burying

Acute OVX resulted in more marbles buried in BIG1 (*post hoc* *t*_(22)_ = 3.248, *p* < 0.004) and BIG2 (*post hoc* *t*_(22)_ = 3.193, *p* < 0.005) females when compared to the sham operated groups (*F*_(1,134)_ = 18.15, *p* < 0.0001). No significant differences were observed between OVX and sham operated SMALL (SML1: *post hoc* *t*_(21)_ = 0.525, *p* > 0.60; SML2: *post hoc* *t*_(22)_ = 1.028, *p* > 0.31) and Control (C1: *post hoc* *t*_(22)_ = 1.732, *p* > 0.09; C2: *post hoc* *t*_(22)_ = 0.650, *p* > 0.52) strains (Figure 1B), which explains the significant strain by treatment interaction effect (*F*_(2,134)_ = 3.49; *p* < 0.034). BIG females buried more marbles than the SMALL females, with the Control mice showing intermediate values (*F*_(2,*3*)_ = 24.24, *p* < 0.015). The replicate nested within strain effect was also significant (*F*_(3,134)_ = 5.56, *p* < 0.0013), predominantly due to the SML1 females burying fewer marbles than the SML2 females (sham: *post hoc* *t*_(21)_ = 2.324, *p* < 0.05; OVX: *post hoc* *t*_(22)_ = 2.922, *p* < 0.008).

### Acute OVX Increased Anxiety-Like Behavior in Compulsive-Like BIG Strains

#### Anxiety-Like Open Field Behavior

**Figure 2:**
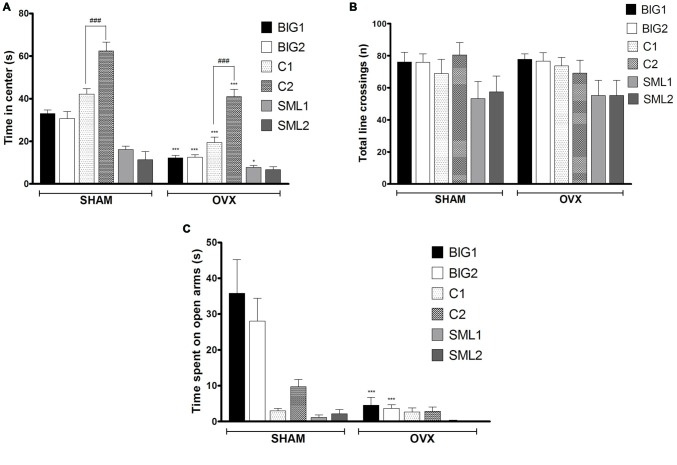
**Anxiety-like behavior in BIG, SMALL and Control strains.** The data represent the mean (± SEM) for **(A)** time spent on center in open field and **(B)** total number of line crossings and **(C)** time spent on open arm in elevated plus maze of the two replicates of the BIG, SMALL and Control strains. ***(*p* < 0.0001) and *(*p* < 0.05) indicates significant differences between sham and OVX groups. ^###^(*p* < 0.0001) indicate significant differences between replicate strains.

In the anxiety-like open field test, the BIG1 (*post hoc* *t*_(22)_ = 5.697, *p* < 0.0001), BIG2 (*post hoc* *t*_(22)_ = 5.008, *p* < 0.0001), C1 (*post hoc* *t*_(22)_ = 6.272, *p* < 0.0001), C2 (*post hoc* *t*_(22)_ = 5.927, *p* < 0.0001), and SML1 (*post hoc* *t*_(21)_ = 2.296, *p* < 0.032) OVX females spent significantly less time in the center when compared to the sham groups (*F*_(1,134)_ = 119.24, *p* < 0.0001). No significant difference was observed between OVX and sham groups in SML2 (*post hoc* *t*_(22)_ = 1.286, *p* > 0.21) females for the time spent in the center (Figure 2A), which explains the significant strain by treatment interaction effect (*F*_(2,134)_ = 10.55; *p* < 0.0015). No significant strain effect was found (*F*_(2,*3*)_ = 6.51, *p* > 0.08), although the Control strains tended to be the least anxious and the SMALL strains the most, while the BIG strains tended to be intermediate. The replicate nested within strain effect was significant (*F*_(3,134)_ = 22.87, *p* < 0.0001), predominantly due to the C1 females spending less time in the center than the C2 females (sham: *post hoc* *t*_(22)_ = 5.582, *p* < 0.0001; OVX: *post hoc* *t*_(22)_ = 5.927, *p* < 0.0001). For total number of line crossings, as a measure of locomotor activity (Figure 2B), no differences were observed among the strains (*F*_(2,3)_ = 0.10, *p* > 0.90) and between sham and OVX groups (*F*_(1,134)_ = 0.03, *p* > 0.80).

#### Anxiety-Like Elevated Plus Maze Behavior

Acute OVX resulted in less time spent on the open arms in the elevated plus maze test for BIG1 (*t*_(22)_ = 6.320, *p* < 0.0001) and BIG2 (*t*_(22)_ = 4.934, *p* < 0.0001) females when compared to the sham groups (*F*_(1,134)_ = 30.14, *p* < 0.0001). No significant differences were observed in Control (C1: *post hoc* *t*_(22)_ = 0.0676, *p* > 0.94 and C2: *post hoc* *t*_(22)_ = 1.403, *p* > 0.17) and SMALL (SML1: *post hoc* *t*_(21)_ = 0.1833, *p* > 0.85 and SML2: *post hoc* *t*_(22)_ = 0.4394, *p* > 0.66) OVX strains when compared to the sham operated mice (Figure 2C), which explains the significant strain by treatment interaction effect (*F*_(2,134)_ = 17.50, *p* < 0.0001). The BIG females spent the most time on the open arms, followed by the Control females, and the SMALL mice showed the highest level of anxiety-like behavior (*F*_(2,134)_ = 26.84; *p* < 0.0001). The replicate nested within strain effect was not significant (*F*_(3,134)_ = 0.86, *p* > 0.46).

### Acute OVX Did Not Affect Recognition Index (RI) for Compulsive-Like BIG Strains in Novel Object Recognition

**Figure 3:**
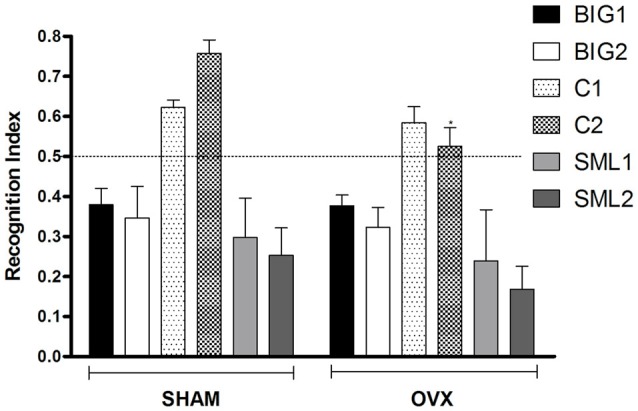
**Novel object recognition in BIG, SMALL and Control strains.** The data represent the mean (± SEM) for the recognition index (RI) in the novel object recognition test between sham and OVX groups of the two replicates of the BIG, SMALL and Control strains. *(*p* < 0.05) indicates significant differences between sham and OVX groups.

The RI was significantly reduced in OVX females compared to sham operated mice (*F*_(1,134)_ = 3.94; *p* < 0.05; Figure 3) with the C2 OVX females having a significantly lower RI than the sham operated C2 mice (*post hoc* *t*_(22)_ = 2.569, *p* < 0.05), while the other strains did not show significant differences (BIG1: *post hoc* *t*_(22)_ = 0.02763, *p* > 0.97; BIG2: *post hoc* *t*_(22)_ = 0.2579, *p* > 0.78; C1: *post hoc* *t*_(22)_ = 0.4236, *p* > 0.66; SML1: *post hoc* *t*_(21)_ = 0.6280, *p* > 0.52; SML2: *post hoc* *t*_(22)_ = 0.9393, *p* > 0.34). The Control females had significantly higher RIs than the BIG and SMALL mice (*F*_(2,134)_ = 37.70; *p* < 0.0001). The replicate nested within strain (*F*_(3,134)_ = 0.53, *p* > 0.66) and the strain by treatment interaction (*F*_(2,134)_ = 0.92, *p* > 0.40) effects were not significant.

### Plasma E2 and P4 Levels Declined in BIG Strains Following Acute OVX

**Figure 4:**
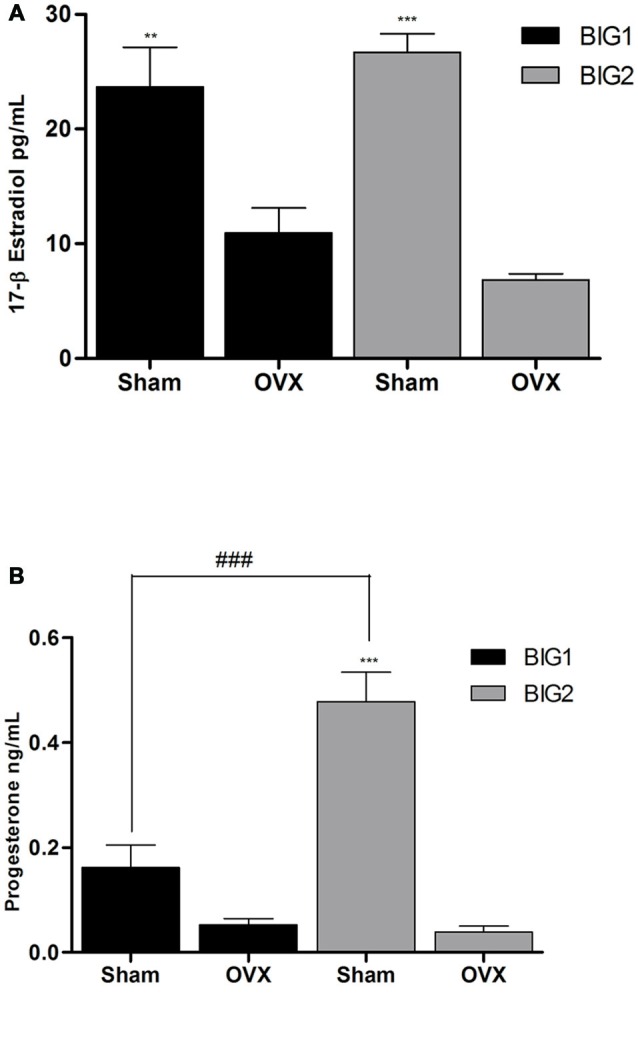
**Ovarian E2 and P4 plasma levels in BIG strains.** The data represent the mean (± SEM) for plasma **(A)** 17β-estradiol (E2) levels and **(B)** progesterone (P4) levels of the BIG1 and BIG2 strains. **(*p* < 0.001) and ***(*p* < 0.0001) indicates significant differences between sham and OVX groups. ^###^(*p* < 0.0001) indicate significant differences between replicate strains.

Plasma E2 levels in acute OVX BIG1 (*post hoc* *t*_(9)_ = 5.501 *p* < 0.0001) and BIG2 (*post hoc* *t*_(10)_ = 6.948 *p* < 0.0001) mice were significantly and similarly (strain: *F*_(1,19)_ = 0.00; *p* > 0.99; strain by treatment interaction: *F*_(1,19)_ = 2.99; *p* > 0.10) reduced when compared to the sham females (treatment: *F*_(1,19)_ = 63.23; *p* < 0.0001; Figure 4).

P4 levels were significantly reduced in the BIG2 (*post hoc* *t*_(12)_ = 8.665 *p* < 0.0001) but not in BIG1 (*post hoc* *t*_(10)_ = 1.993 *p* > 0.058) OVX females when compared to their sham counterparts (*F*_(1,19)_ = 61.17; *p* < 0.0001; Figure 4), which explains the significant strain (*F*_(1,19)_ = 17.30; *p* < 0.0005) and strain by treatment interaction (*F*_(1,19)_ = 18.93; *p* < 0.0004) effects.

### Acute E2 and P4 Administration in OVX Female Mice

#### Compulsive-Like Nesting Was Attenuated by E2 But Not P4 Administration

**Figure 5:**
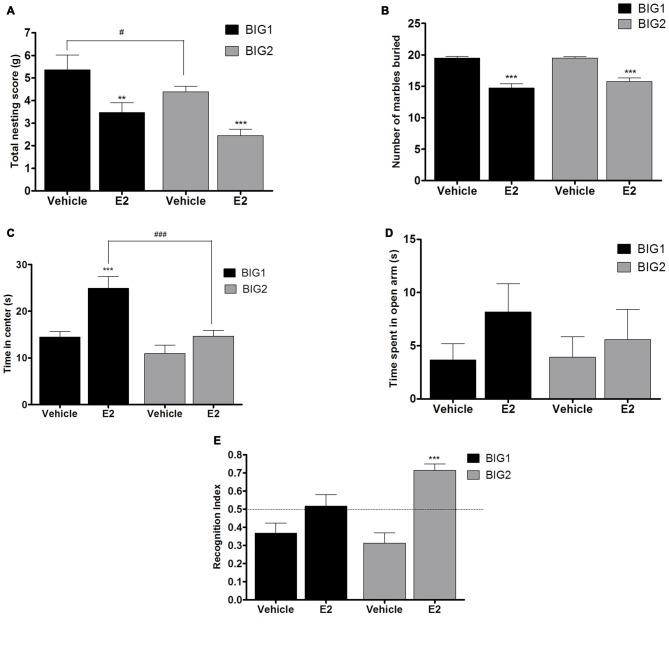
**E2 administration in OVX mice.** The data represent the mean (± SEM) for **(A)** nesting score in grams, **(B)** number of marbles buried, **(C)** time in center in open field, **(D)** time spent on open arms in elevated plus maze and **(E)** RI in novel object recognition of the BIG1 and BIG2 strains. **(*p* < 0.001) and ***(*p* < 0.0001) indicates significant differences between vehicle and E2 treatment groups. ^#^(*p* < 0.05) and ^###^(*p* < 0.0001) indicate significant differences between replicate strains.

Acute E2 administration resulted in a significant and similar (strain by treatment interaction: *F*_(1,43)_ = 0.00, *p* > 0.94) decline of nesting scores in the BIG1 (*post hoc* *t*_(22)_ = 3.000, *p* < 0.007) and BIG2 (*post hoc* *t*_(22)_ = 3.814, *p* < 0.001) OVX females when compared to the vehicle controls (*F*_(1,43)_ = 19.51; *p* < 0.0001; Figure 5A). The BIG1 females used more cotton for their nest compared to the BIG2 mice (*F*_(1,43)_ = 5.69, *p* < 0.022), which replicated the results in Figure 1A.

**Figure 6:**
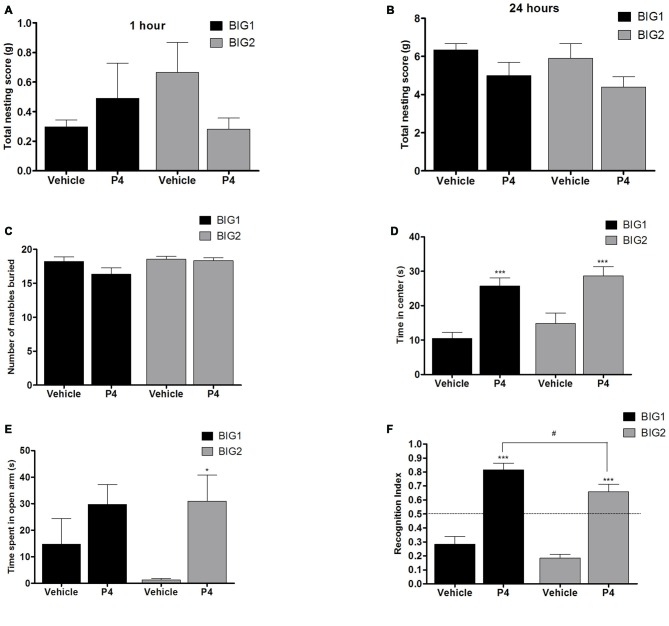
**P4 administration in OVX mice.** The data represent the mean (± SEM) for **(A)** nesting score in grams between 0–1 h, **(B)** nesting score in grams between 0–24 h, **(C)** number of marbles buried, **(D)** time in center in open field, **(E)** time spent on open arms in elevated plus maze and **(F)** RI in novel object recognition of the BIG1 and BIG2 strains. *(*p* < 0.05) and ***(*p* < 0.0001) indicates significant differences between vehicle and P4 treatment groups. ^#^(*p* < 0.05) indicate significant differences between replicate strains.

No significant differences were observed in the 1 h (*F*_(1,32)_ = 0.96; *p* > 0.33) and 24 h (*F*_(1,32)_ = 3.47; *p* > 0.05) nesting scores of acute P4 treated BIG1 and BIG2 OVX females when compared to the vehicle treated controls (Figures 6A,B). For 1 h and 24 h nesting scores, the strain (*F*_(1,32)_ = 0.37; *p* > 0.54; *F*_(1,32)_ = 1.05; *p* > 0.31, respectively) and strain by treatment interaction (*F*_(1,32)_ = 2.06; *p* > 0.16; *F*_(1,32)_ = 0.00; *p* > 0.95, respectively) effects were not significant.

#### Compulsive-Like Marble Burying was Attenuated by E2 But Not P4 Administration

BIG1 (*post hoc* *t*_(22)_ = 6.447, *p* < 0.0001) and BIG2 (*post hoc* *t*_(22)_ = 7.606, *p* < 0.0001) OVX females buried significantly less marbles in the acute E2 treatment group when compared to the vehicle groups (*F*_(1,43)_ = 85.67, *p* < 0.0001; Figure 5B). The strain (*F*_(1,43)_ = 0.79, *p* > 0.37) and strain by treatment interaction (*F*_(1,43)_ = 1.24, *p* > 0.27) effects were not significant, indicating that the BIG1 and BIG2 OVX females had similar marble burying scores and responses to E2.

P4 administration did not cause significant changes in the number of marbles buried by BIG1 and BIG2 OVX females when compared to the vehicle control mice (*F*_(1,32)_ = 2.64; *p* > 0.11; Figure 6C). The strain (*F*_(1,32)_ = 3.22, *p* > 0.08) and strain by treatment interaction (*F*_(1,32)_ = 1.64, *p* > 0.20) effects were also not significant.

#### E2 and P4 Treatment Showed Strain Dependent Decreases in Anxiety-Like Behavior in the Open Field

In the acute E2 administration group, the BIG1 OVX females (*post hoc* *t*_(22)_ = 4.245, *p* < 0.0005) spent more time in the center when compared to the vehicle group (*F*_(1,43)_ = 16.70, *p* < 0.0002; Figure 5C). No significant difference in the time spent in the center was observed between E2 and vehicle groups in BIG2 OVX females (*post hoc* *t*_(22)_ = 1.564 *p* > 0.13). The significant strain effect (*F*_(1,43)_ = 17.05; *p* < 0.0003) was due to BIG1 E2 administered females performing better than the BIG2 E2 administered females (*post hoc* *t*_(22)_ = 4.178, *p* < 0.0005). The strain by treatment interaction effect was not significant (*F*_(1,43)_ = 3.77, *P* > 0.058), which showed that the BIG2 females responded to E2 in a similar direction as the BIG1 females.

P4 administered groups in both BIG1 (*post hoc* *t*_(16)_ = 4.311, *p* < 0.0005) and BIG2 (*post hoc* *t*_(16)_ = 3.904, *p* < 0.001) OVX females spent significantly more time in the center when compared to their respective vehicle control groups (*F*_(1,32)_ = 33.75; *p* < 0.0001; Figure 6D). The strain (*F*_(1,32)_ = 2.17, *p* > 0.15) and strain by treatment interaction (*F*_(1,32)_ = 0.08, *p* > 0.77) effects were not significant, indicating that the BIG1 and BIG2 OVX females spent similar times in the center and responded similarly to P4.

#### P4, But Not E2, Treatment Had an Effect on Anxiety-Like Elevated Plus Maze Behavior

No significant differences in the time spent on the open arm in the elevated plus maze test was observed between E2 and vehicle groups of BIG1 and BIG2 OVX females (*F*_(1,43)_ = 1.81; *p* > 0.18; Figure 5D). In addition, the strain (*F*_(1,43)_ = 0.22, *p* > 0.63) and strain by treatment interaction (*F*_(1,43)_ = 0.39, *p* > 0.53) effects were also not significant.

Overall, P4 administration significantly increased the time spent on the open arms compared to the vehicle groups (*F*_(1,32)_ = 8.20, *p* < 0.0073; Figure 6E), which was significant when comparing the BIG2 P4 treated to the BIG2 vehicle OVX females (*post hoc* *t*_(16)_ = 2.692, *p* < 0.014). Although the trend was in the same direction as the BIG2 OVX females, no significant difference was observed between P4 and vehicle groups of BIG1 OVX females (*post hoc* *t*_(16)_ = 1.358, *p* > 0.18). The strain (*F*_(1,32)_ = 0.63, *p* > 0.43) and strain by treatment interaction (*F*_(1,32)_ = 0.89, *p* > 0.35) effects were not significant, indicating that the BIG1 and BIG2 OVX females spent similar times on the open arms, and the BIG1 females responded to P4 in a similar direction as the BIG2 females.

#### E2 and P4 Improved Recognition Index (RI) in Object Recognition Memory With a Replicate Effect Seen in E2 Treatment

Overall, E2 administration significantly increased performance in the novel object recognition test compared to the vehicle treated OVX females (*F*_(1,43)_ = 26.95, *p* < 0.0001; Figure 5E), which was significant for the BIG2 OVX females compared to their vehicle treated counterparts (*post hoc* *t*_(22)_ = 5.358, *p* < 0.0001). Although the trend was in the same direction as the BIG2 OVX females, no significant difference was observed in the RI of E2 treated BIG1 OVX females compared to vehicle treated females (*post hoc* *t*_(22)_ = 1.946, *p* > 0.06), which explains the significant strain by treatment interaction effect (*F*_(1,43)_ = 5.55, *p* < 0.024). The strain effect was not significant (*F*_(1,43)_ = 1.93, *p* > 0.17), which indicates that the BIG1 and BIG2 females had overall similar memory scores.

P4 administration enhanced the performance in the novel object recognition test for both the BIG1 (*post hoc* *t*_(16)_ = 3.855, *p* < 0.001) and the BIG2 (*post hoc* *t*_(16)_ = 4.726, *p* < 0.0001) OVX females compared to their vehicle control groups (*F*_(1,32)_ = 114.3; *p* < 0.0001; Figure 6F). The BIG1 OVX females had higher RIs compared to the BIG2 OVX females (strain: *F*_(1,32)_ = 7.32; *p* < 0.011, irrespective of treatment group (strain by treatment interaction: *F*_(1,32)_ = 0.36; *p* > 0.55)).

## Discussion

In the current study we showed that acute OVX for 7 days resulted in a significant increase in the compulsive-like nesting and marble burying behaviors of BIG1 and BIG2 female mice. No increase in nesting and marble burying was observed for the Control and SMALL strains, which shows the specificity of the OVX effects for the compulsive-like condition. The exacerbations in compulsive-like behaviors in BIG mice were attenuated by acute subcutaneous administration of E2, but not P4. Human studies have shown that gonadal steroids trigger or precipitate mood disorders in women with a history of an already existing disease condition when compared to women without it (Hay et al., 1994; Schmidt et al., 1998; Clayton and Ninan, 2010). Onset and exacerbation of OCD associated with pregnancy and postpartum has been shown in human studies (Neziroglu et al., 1992; Williams and Koran, 1997; Labad et al., 2005; Uguz et al., 2007; Forray et al., 2010) establishing a strong link between reproductive events and OCD. However, there is lack of literature on how obsessions and compulsions and associated affective and cognitive behaviors vary during induced menopause. According to a review (Forray et al., 2010) a large variation exists in human studies on onset and exacerbation of OCD during reproductive events and one of the contributing factors could be innate differences in patient populations. In congruence with this we found that BIG1 sham females had higher nesting scores when compared to BIG2 sham females. This variation in compulsive-like nesting behavior was also seen post acute OVX but was abolished in the E2 treatment regimen. What is more intriguing is the fact that BIG1 and BIG2 females did not exhibit variation in compulsive-like marble burying. This is an interesting finding indicating heterogeneity in the BIG strains based on compulsive-like traits and genetic background as often seen in subgroups of OCD patients (Fontenelle et al., 2005; Grados and Riddle, 2008; Leckman et al., 2009). Whether E2 might be more effective compared to P4 in reducing OCD symptoms in postmenopausal females, as seen in our OVX compulsive-like mice, remains to be elucidated.

A prior study has shown that acute E2 administration along with P4 to OVX rats reduced compulsive perseverance in the T-maze (Fernández-Guasti et al., 2006). This is similar to our findings in the compulsive-like mice though, E2 alone had an attenuating effect in the OVX state. Interestingly, previous findings show that acute P4 administration reduced compulsive-like marble burying behavior in male rats (Umathe et al., 2009). However, in the current study we did not see an anti-compulsive effect of P4 treatment in BIG mice. This could be due to various factors, including using mouse strains and females in our study compared to male rats in the (Umathe et al., 2009) study.

In the anxiety-like measures, the OVX BIG strains spent less time in the center of the open field and also explored the open arm less in elevated plus maze when compared to the sham groups. The SMALL and the Control OVX strains showed no significant changes in open field and elevated plus maze when compared to their sham counterparts. Therefore, OVX worsened anxiety-like behaviors in compulsive-like condition specifically. Acute administration of E2 resulted in increased time spent in the central square of the open field in BIG1, but not BIG2 strains, indicating a strain dependent effect in the E2 treatment response. The acute dosage and the time frame of administration of E2 in our study previously also showed anxiolytic effects in the open field in acutely OVX mice (Walf et al., 2008b) and rats (Walf and Frye, 2009). In the plus maze no significant effect of E2 was observed in the compulsive-like mice, which could be due to the fact that the open field and the elevated plus maze tests measure different aspects of emotionality associated with anxiety (Ramos, 2008; Anchan et al., 2014).

P4 treatment, on the other hand, decreased anxiety-like behavior in open field for both BIG1 and BIG2 strains. For the elevated plus maze, however, P4 was effective only in the BIG2 and not the BIG1 strain. These results indicate a strain specific response to anxiety-like behavior due to P4 administration. Strain specific effects of E2 and P4 on behavioral responses have been sparsely explored in rodent studies. Only one study has shown significant strain specific effects of E2 on depressive-like forced swim behavior (Koss et al., 2012). Behavioral responses to alterations in gonadal steroids have been found to vary in women with and without premenstrual syndrome (Schmidt et al., 1998), which might be similar to the differences in behavioral responses to E2 and P4 in the BIG1 and BIG2 strains.

The association of memory impairment with OCD is not clear. Many clinical studies have failed to find any evidence that OCD is associated with memory deficits (McDonald, 1991; Dirson et al., 1995; Radomsky and Rachman, 1999). In addition, no impairment in declarative and short-term memory has been found in OCD patients compared to normal controls (Roth et al., 2004; Demeter et al., 2013). However, many others have reported working memory impairments in patients (Martin et al., 1995; Nakao et al., 2009). Our BIG mice showed a larger object recognition memory deficit than the Control mice, both in the sham and OVX groups. However, the SMALL mice also showed a similar memory deficit and, therefore, whether this memory deficit in the BIG mice was due to a genetic correlation between compulsive-like behaviors and object recognition memory, or was due to genetic differences between the two strains caused by founder effects or random drift (Bult and Lynch, 1996, 2000), remains to be elucidated. Also as the BIG and SMALL strains had a RI below 0.5 they appeared to avoid the new object, while the control mice had an index above 0.5 and appeared to favor the new object.

Acute OVX did not result in significant object recognition memory impairment in the compulsive-like condition. Contradictory evidence exists as to whether acute OVX leads to object recognition memory impairment in normal mice (c57 strain). While few studies have shown memory loss during acute OVX (Gresack and Frick, 2006; Rhodes and Frye, 2006), others show the opposite (Willard et al., 2011; Fonseca et al., 2013; Bastos et al., 2015). However, chronic OVX has consistently caused poor performance in the novel object recognition test (Fonseca et al., 2013; Bastos et al., 2015). Though there was no overall effect in novel object recognition memory among sham and OVX strains, E2 treatment improved the RI in only the BIG2 but not the BIG1 strain. P4 treatment however improved the RI in both the strains. In the current context of investigation object recognition was performed to evaluate short term or working memory impairments associated with OCD in the mouse strains. However, object recognition encompasses just one aspect of assessing otherwise very complex memory consolidation and cognition process in rodents. In future studies we aim to include a more robust assessment of both short-term and spatial memory components.

The current study supports a complex interplay of genetic background and sex steroids during acute ovarian dysfunction in the compulsive-like condition. We report exacerbation of compulsive-like behaviors with trait specific intra-strain variation during acute ovarian failure in the spontaneously compulsive-like mouse strains only, which was restored by E2 and not P4. This effect was similar for the spontaneously compulsive-like BIG1 and BIG2 strains, unlike the associated anxiety-like and cognitive-like behaviors, which displayed differences between the BIG1 and BIG2 strains for both E2 and P4 effects on these behaviors. We therefore hypothesize that the associated comorbidities in the surgical menopause state in the OCD condition might vary among individuals because of genetic differences. Future studies will focus on investigating effects of E2 and P4 on the chronic OVX state and also the potential signaling pathways in the brain of compulsive-like mice. Overall, the results presented here strengthen the face, predictive and construct validities of the mouse model for investigating heterogeneity associated with OCD during ovarian failures in females.

## Author Contributions

SM, CPB and KB conducted all experiments and performed data analysis. SM and CPB lead manuscript writing efforts. AB-I and GSP made significant contributions to research design, data interpretations and manuscript preparation.

## Funding

Research reported in this publication was supported by an Institutional Development Award (IDeA) from the National Institute of General Medical Sciences of the National Institutes of Health under grant number P20GM103395 to SM. The content is solely the responsibility of the authors and does not necessarily reflect the official views of the NIH. The work was also supported by College of Natural Sciences and Mathematics (CNSM) and the Office of the Vice-Chancellor for Research to SM and AB-I. CPB received a fellowship from CAPES/Brazil for this project. These funding sources did not have a role in the study design, data collection, analysis, interpretation and submission of this article for publication.

## Conflict of Interest Statement

The authors declare that the research was conducted in the absence of any commercial or financial relationships that could be construed as a potential conflict of interest.
